# The ATXN2 9 bp duplication in SCA3: clarifying evidence and correcting misinterpretations

**DOI:** 10.1186/s40478-026-02305-y

**Published:** 2026-05-01

**Authors:** Jose Miguel Laffita-Mesa, Martin Paucar, Per Svenningsson

**Affiliations:** 1 Department of Clinical Neuroscience, Karolinska Institutet, Stockholm, Sweden; 2 Department of Neurobiology, Care Sciences and Society, Karolinska Institutet, Stockholm, Sweden

**Keywords:** ATXN2, SCA3, Variants, 9 bp duplication, Biomarkers, Gene modifier, CAG repeats

## Abstract

Lauerer et al. (Acta Neuropathol Commun 13:157, 2025) recently investigated the role of *ATXN2* variants, including intermediate CAG repeats and a 9-bp duplication, in spinocerebellar ataxia type 3 (SCA3). While their study contributes valuable data, several of their interpretations diverge from our previous findings and require clarification. Our original hypothesis was not that the duplication alone modified age at onset, but that its effect emerges in combination with an intermediate-length *ATXN2* allele (29 CAGs). Their claim of a protective effect is unsupported by non-significant results, which cannot establish equivalence. Furthermore, evidence from multiple studies demonstrates that both ATG1 and ATG2 can initiate translation, with redundancy explaining the in vivo ~ 140–145 kDa *ATXN2* protein, thereby countering the assertion that the duplication is relevant only at the DNA level. We also correct the misattribution of our segregation analyses and emphasize that low frequency does not negate biological significance, as rare variants can act as genetic modifiers. Taken together, convergent DNA, RNA, and protein evidence supports the relevance of the *ATXN2* 9-bp duplication as part of the broader network of modifiers in SCA3.

## Introduction

We read with interest the recent article by Lauerer et al. [1], which examined the role of *ATXN2* variants—including intermediate CAG repeats and a 9 bp duplication, in modulating the clinical course of SCA3. Their work represents an important contribution to ongoing efforts to disentangle the complexity of genetic modifiers in neurodegeneration, particularly in spinocerebellar ataxia type 3 (SCA3). The study was conducted in one of the largest SCA3 cohorts by an excellent consortium. However, while we acknowledge the value of their data, several of their interpretations diverge from, or misrepresent, our previous findings. Specifically, four points require clarification: *(i) the scope of our original hypothesis regarding the 9 bp duplication*,* (ii) the claim of a protective effect on age at onset*,* (iii) the assertion that this variant is relevant only at the DNA level*,* and (iv) the misrepresentation of our segregation analyses*.

## Central contention

(i) Our hypothesis was never that the 9 bp duplication alone altered age at onset (AO), as no such cases were present in our SCA3 casuistic. Rather, we argued, in a dialectical sense, that the modulatory effect arises from the interaction of two elements: the 9 bp duplication and an intermediate-length *ATXN2* allele (29 CAGs). Only this combination was associated with a significant reduction in AO in our cohort, both in SCA3 and in C9-ALS. Notably, the only subjects carrying the 9 bp duplication alone were Parkinson’s disease patients, where no effect was observed, consistent with ataxin-2 being less central to that disorder. By their own admission, such cases were absent from their cohort; thus, their data cannot refute our model [1].

In logical terms if:

- 22CAG + 9 bp-dup → no ↓AO (Lauerer et al. [1])
- 29CAG + 9 bp-dup → ↓AO (Laffita-Mesa et al. [2])

Concluding non-replication by denying the antecedent constitutes a logical fallacy. Importantly, their own observation of faster progression in SCA3 carriers with a 9 bp duplication remains compatible with our interpretation of a modulatory role.

(ii) Lauerer et al. reported a non-significant increase in AO and extended this to claim an “opposite” (protective) effect. Yet a non-significant result cannot establish protection. Assuming that “not worsening = protective” is a logical fallacy: the variant may be neutral, or the study underpowered to detect effects. Absence of evidence is not evidence of absence, and demonstrating protection would require a prespecified analysis, a Bayesian framework, or an equivalence/non-inferiority test, none of which were applied. Thus, their claim of a protective effect is not supported by the statistical framework applied.
(iii) The authors further claimed that the duplication is relevant only at the DNA level because it is not expressed at RNA level. This argument relates to the ATG initiation debate. In GRCh38/hg38, ATG1 lies ~ 496 bp upstream of the CAG and ATG2 only 12 bp upstream. Yet across multiple antibodies, including the one used in their own study, the predominant band corresponds to ~ 140 kDa, consistent with ATG1 initiation. If translation initiated at ATG2, the protein would be ~ 17 kDa smaller (~ 124 kDa) than the full-length isoform.

Chronologically, Aguiar et al. [3, 4] demonstrated preferential ATG1 usage. Scoles et al. [5] concluded ATG2 necessity from luciferase constructs, but this was context-specific and does not explain the in vivo ~ 140 kDa protein. Without a double-mutant construct, redundancy cannot be excluded. Chitre and Emery [6] reconciled these discrepancies by showing both ATGs are utilized, each undergoing proteolytic processing, and confirming by knockdown that the ~ 145 kDa protein corresponds to endogenous *ATXN2*. Thus, ATG1 promotes the full-length isoform, while ATG2 usage is not mutually exclusive with functionality.

Supporting this, Inada et al. [7] described a 21-bp duplication between ATG1 and ATG2, which produced aggregation and was detected by 1C2 antibody even without an expanded repeat. This reinforces the plausibility of small duplications near start codons producing protein-level effects mimicking polyQ pathology and at the same support ATG1 usage in producing pathological ataxin-2.

Our own work further confirmed in vivo expression of the duplication using ddPCR in blood and lymphoblastoid cells targeting rs695871, the same SNP used for co-segregation. This demonstrates transcription and incorporation into protein.

### Additional clarifications

(iv)  Lauerer et al. also misrepresented our work by claiming that we reported co-segregation with rs7969300. This is incorrect. Our analyses were based on rs695871 in *ATXN2* exon 1 (~ 200 bp downstream of the duplication; ENST00000550104.5), not on rs7969300 in exon 2 (~ 43.757 Kb downstream) [2].

Finally, dismissing the 9-bp duplication as irrelevant due to low frequency is premature. Rare variants can act as meaningful modifiers. For example, Weber et al. [8] showed that *PRKN* rs1801582 C/C genotype (3.1%) influences AO in SCA3, a frequency comparable to the 9-bp duplication (4.18%). In rare diseases, broad effects are difficult to prove, yet their biological significance remains.

## Conclusion

Rather than being dismissed, this variant should be considered within a broader network of modifiers supported by convergent evidence at the DNA, RNA, and protein levels. A more integrative and dialectical perspective will better serve the field, fostering rigorous dialogue, accurate attribution, and steady progress toward biomarker discovery and therapeutic innovation.

## Data Availability

No datasets were generated or analysed during the current study.
